# Supplementary material for: Individual differences drive social hierarchies in male mouse societies
Source: eLife. 2026 Sep 1;15:RP109354. doi: 10.7554/eLife.109354 (PMC13533481; doi:10.7554/eLife.109354)
Supplement: Supplementary file 2 [file elife-109354-repstand1.pdf]

NOTE: Please save this file locally before filling in the table, DO NOT work on the file within your internet browser as changes will not be saved. Adobe Acrobat Reader (available free here) is recommended for completion.

The ARRIVE guidelines 2.0: author checklist

The ARRIVE Essential 10

These items are the basic minimum to include in a manuscript. Without this information, readers and reviewers cannot assess the reliability of the findings.

Item		Recommendation	Section/line number, or reason for not reporting
**Study design**	1	For each experiment, provide brief details of study design including:	
		a. The groups being compared, including control groups. If no control group has been used, the rationale should be stated.	Results: Ecological longitudinal assessment in the NoSeMaze + Fig. 1<br>Methods: Animals, Non-invasive sensor-rich maze (NoSeMaze) and behavioral data acquisition
		b. The experimental unit (e.g. a single animal, litter, or cage of animals).	Methods: Animals, Non-invasive sensor-rich maze (NoSeMaze) and behavioral data acquisition
**Sample size**	2	a. Specify the exact number of experimental units allocated to each group, and the total number in each experiment. Also indicate the total number of animals used.	Supplementary Table S1 and S2<br>Methods: Animals, Non-invasive sensor-rich maze (NoSeMaze) and behavioral data acquisition
		b. Explain how the sample size was decided. Provide details of any *a priori* sample size calculation, if done.	No formal a priori sample size calculation was performed, as this study was exploratory in nature and aimed to generate a comprehensive behavioral dataset across multiple social contexts. Instead, sample size
**Inclusion and exclusion criteria**	3	a. Describe any criteria used for including and excluding animals (or experimental units) during the experiment, and data points during the analysis. Specify if these criteria were established *a priori*. If no criteria were set, state this explicitly.	Reinforcement learning: Line 725-726, Line 175, Line 684; Supplementary Table S2;<br>Tube-based metrics: Line 256, Line 684; Supplementary Table S2;
		b. For each experimental group, report any animals, experimental units or data points not included in the analysis and explain why. If there were no exclusions, state so.	Exclusion reported in Supplementary Table S2 in detail. Problems with the reinforcement learning paradigm in group 12-15 and with the RFID detectors (antennas) in group 16, 20, 21
		c. For each analysis, report the exact value of *n* in each experimental group.	In the respective figures, all data points are plotted, if possible. Source data contains exact information on each n.
**Randomisation**	4	a. State whether randomisation was used to allocate experimental units to control and treatment groups. If done, provide the method used to generate the randomisation sequence.	Line 149-152; Line 665-667; Randomization of group allocation (NoSeMazes) and home cages
		b. Describe the strategy used to minimise potential confounders such as the order of treatments and measurements, or animal/cage location. If confounders were not controlled, state this explicitly.	Blinding and randomization, see also Line 149-152; Line 665-667;
**Blinding**	5	Describe who was aware of the group allocation at the different stages of the experiment (during the allocation, the conduct of the experiment, the outcome assessment, and the data analysis).	Line 149-152; Line 665-667; For each three-week NoSeMaze round, animals were allocated to specific NoSeMazes and group compositions by random permutation of subject IDs in MATLAB, targeting groups of 9 – 10. Group assignments were generated by an investigator not involved in data analysis, and all downstream analyses were
**Outcome measures**	6	a. Clearly define all outcome measures assessed (e.g. cell death, molecular markers, or behavioural changes).	Line 721 ff., Quantification of individual reward-seeking behavioral metrics<br>Line 780 ff., Quantification of individual social behavioral metrics
		b. For hypothesis-testing studies, specify the primary outcome measure, i.e. the outcome measure that was used to determine the sample size.	Line 721 ff., Quantification of individual reward-seeking behavioral metrics<br>Line 780 ff., Quantification of individual social behavioral metrics
**Statistical methods**	7	a. Provide details of the statistical methods used for each analysis, including software used.	Statistical analysis: Line 862 ff.<br>For metric calculation:<br>Line 721 ff., Quantification of individual reward-seeking behavioral metrics<br>Line 780 ff., Quantification of individual social
		b. Describe any methods used to assess whether the data met the assumptions of the statistical approach, and what was done if the assumptions were not met.	Statistical analysis: Line 862 ff.;<br>Cross-validation with LMEs<br>For metric calculation:<br>Line 721 ff., Quantification of individual reward-seeking behavioral metrics
**Experimental animals**	8	a. Provide species-appropriate details of the animals used, including species, strain and substrain, sex, age or developmental stage, and, if relevant, weight.	Line 647 ff.; Animals (including strain, sex, age, housing, genotype)<br>Supplementary Table S1 (for every animal)
		b. Provide further relevant information on the provenance of animals, health/immune status, genetic modification status, genotype, and any previous procedures.	Line 647 ff.; Animals (including strain, sex, age, housing)<br>Supplementary Table S1
**Experimental procedures**	9	For each experimental group, including controls, describe the procedures in enough detail to allow others to replicate them, including:	
		a. What was done, how it was done and what was used.	Methods Line 646 ff.; Supplementary Table S1 and S2
		b. When and how often.	Methods Line 646 ff.; Supplementary Table S1 and S2
		c. Where (including detail of any acclimatisation periods).	Methods Line 646 ff.; Supplementary Table S1 and S2
		d. Why (provide rationale for procedures).	Methods Line 646 ff.; Supplementary Table S1 and S2
**Results**	10	For each experiment conducted, including independent replications, report:	
		a. Summary/descriptive statistics for each experimental group, with a measure of variability where applicable (e.g. mean and SD, or median and range).	Summary and descriptive statistics are reported in the main and supplemental figures, respectively. See also 'source data', 'source data supplement', 'extended data', and 'extended data supplement'
		b. If applicable, the effect size with a confidence interval.	Confidence intervals are reported in each main figure. Procedures in the Methods (Line 891 ff.)
