# Supplementary material for: Individual differences drive social hierarchies in male mouse societies
Source: eLife. 2026 Sep 1;15:RP109354. doi: 10.7554/eLife.109354 (PMC13533481; doi:10.7554/eLife.109354)
Supplement: Peer review history

# Editor report: eLife Assessment

**DOI:** 10.7554/eLife.109354.3.sa0

**Authors:** Gordon J Berman

**Affiliations:** Emory University United States

**Keywords:** Compelling, Important

This **important** study introduces NoSeMaze, a semi-naturalistic platform for continuous, high-dimensional tracking of social and cognitive behaviors in group-housed mice, and uses it to show that individual social rank is stable across changing social contexts. By integrating automated dominance measures, proactive social behaviors, and reinforcement-learning-based profiles, the authors demonstrate a novel framework for examining how stable individual differences shape social structure. The findings provide **compelling** evidence that dominance traits are stable across changing social contexts and largely independent of non-social cognitive performance, supporting the view that social rank reflects an intrinsic dimension of individuality. However, the broader functional significance of dominance in this paradigm remains somewhat ambiguous, including the extent to which the measured behaviors capture how dominance operates in more naturalistic social settings. This work will be of broad relevance for behavioral neuroscience and social behavior research.

# Referee report: Reviewer #2 (Public review)

**DOI:** 10.7554/eLife.109354.3.sa1

Summary:

This manuscript presents the "NoSeMaze", a novel automated platform for studying social behavior and cognitive performance in group-housed male mice. The authors report that mice form robust, transitive dominance hierarchies in this environment and that individual social rank remains largely stable across multiple group compositions. They further demonstrate that social dominance and aggressive behaviors, like chasing, are partially dissociable and that dominance traits are independent of non-social cognitive performance. The study includes a genetic manipulation of oxytocin receptor expression in the anterior olfactory nucleus, which showed only transient effects on social rank.

Strengths:

(1) Innovative Methodology:

The NoSeMaze platform is a technically elegant and conceptually well-integrated system that enables fully automated, long-term monitoring of both social and cognitive behaviors in large groups of group-housed mice. It combines tube-test-like dominance contests, voluntary chase-escape interactions, and an embedded operant olfactory discrimination task within a single, ethologically relevant environment. This modular design allows for high-throughput, minimally invasive behavioral assessment without the need for repeated handling or artificial isolation.

(2) Experimental Scale and Rigor:

The study includes 79 male mice and over 4,000 mouse-days of observation across multiple group reshufflings. The use of RFID-based identification, automated data logging, and longitudinal design enables robust quantification of individual trait stability and group-level social structure.

(3) Multidimensional Behavioral Profiling:

The integration of social (tube dominance, proactive chasing), physical (body weight), and cognitive (olfactory learning task) measures offers a rich, multi-dimensional profile of each individual mouse. The authors' finding that social dominance traits and non-social cognitive performance are largely uncorrelated reinforces emerging models of orthogonal behavioral trait axes or "animal personalities".

(4) Clarity and Data Analysis:

The analytical framework is well-suited to the study's complexity, with appropriate use of dominance metrics, mixed-effects models, and permutation tests. The analyses are clearly explained, statistically rigorous, and supported by transparent supplementary materials.

Weaknesses:

(1) Scope Limitations (Sex):

The study is limited to male mice, which represents a common but problematic bias.

(2) Ambiguity of Dominance as a Construct:

While the study robustly quantifies social rank and hierarchy structure, the broader functional meaning of "dominance" remains unclear.

# Author comment: Author response

**DOI:** 10.7554/eLife.109354.3.sa2

**Authors:** Jonathan Reinwald, Sarah Ghanayem, David Wolf, Max Scheller, Julia Lebedeva, Philipp Lebhardt, Oliver Gölz, Corentin Nelias, Wolfgang Kelsch

**Affiliations:** University Medical Center of the Johannes Gutenberg University Mainz Mainz Germany; University Medical Center of the Johannes Gutenberg University Mainz Mainz Germany; University Medical Center of the Johannes Gutenberg University Mainz Mainz Germany; University Medical Center of the Johannes Gutenberg University Mainz Mainz Germany; University Medical Center of the Johannes Gutenberg University Mainz Mainz Germany; Fraunhofer Institute for Manufacturing Engineering and Automation Mannheim Germany; Fraunhofer Institute for Manufacturing Engineering and Automation Mannheim Germany; University Medical Center of the Johannes Gutenberg University Mainz Mainz Germany; University Medical Center of the Johannes Gutenberg University Mainz Mainz Germany

The following is the authors’ response to the original reviews.

**Public Reviews:**

**Reviewer #1 (Public review):**

Summary:

The goal of the study was to address the question of the degree to which social position in a group is a stable trait that persists across conditions. Reinwald et al. use a custom-built cage system with automated tracking and continuous testing for social dominance that does not require intervention by the experimenter. Remixing of individuals from different groups revealed that social position was rather stable and not really predictable from other measures that were taken. The authors conclude that social position is multifaceted but dependent on characteristics like personality traits.

Strengths:

(1) Reductionistic, highly controlled setting that allows for the control of many confounding variables.

(2) Very interesting and important question.

(3) Confirms the emergence of inter-individual behavior-driven differences in inbred mice in a shared environment.

(4) Innovative paradigm and experimental setup.

(5) Fresh perspective on an old question that makes the best use of modern technology.

(6) Intelligent use of behavioral and cognitive covariables to generate a non-social context.

(7) Bold and almost provocative conclusion, inviting discussion and further elaboration.

We thank Reviewer #1 for this constructive and balanced evaluation of our work, and for highlighting both the conceptual importance of the question and the strengths of our automated, highly controlled approach.

Weaknesses:

(1) Reductionistic, highly controlled setting that blends out much of the complexity of social behavior in a community.

Our goal in developing the NoSeMaze was to provide an enriched yet standardized environment that allows animals to interact freely in a complex setting where arena geometry and access contingencies are held constant across groups. As the Reviewer also notes as a strength, this highly controlled setting with minimal experimenter interference enables us to minimize confounds and provides reproducibility across rounds and groups. We now clarify this explicitly and frame the design as a trade-off between ecological complexity and experimental controllability.

The NoSeMaze is designed as an open system that can incorporate additional configurations. In this first study with the system, we intentionally used a design that focused on single-sex groups without mating, no intruders or external threats, stable environmental conditions, and adult mice. This allowed us to establish social behavior under one defined condition. From here, future studies can add certain levels of ecological and social complexity to progressively understand their impact on specific behaviors and group dynamics.

Accordingly, we have revised the manuscript to clarify the scope and the role of social and environmental conditions to the here observed phenomena. We also describe how future studies with systematically modified conditions can be used to understand how they change behaviors. We further replaced potentially over-broad terms (e.g., “naturalistic,” “real-world”) with more precise wording throughout.

We modified the following sections:

Abstract

We removed “… within naturalistic mouse groups.” (ll. 52-54) and changed the sentence to “The approach thus enables longitudinal modeling of individuality and social position as key resilience factors.”

We changed “… in naturalistic groups.” (l. 38) to “… in larger male mouse groups.” and “… from naturalistic tube competitions …” (ll. 41-42) to “… from incidental competitions in the integrated tube tests …”. We also deleted “naturalistic” in l. 50.

Discussion (ll. 691-701)

“…The NoSeMaze aims to increase environmental complexity and group dynamics while retaining experimental control, enabling longitudinal high-dimensional phenotyping of individuals. At the same time, it is a controlled laboratory group-housing habitat optimized to capture a subset of the determinants of social complexity present in natural communities. The strength of this design lies in the continuous, observer-independent observation of complex behaviors in defined environmental and social contexts. Accordingly, we interpret our findings as applying to the social contexts and environmental conditions tested here. Building on this, its modular design allows for introducing additional environmental and social factors like stressors, mating behavior, or resource competition in the future to understand their respective impact in modifying social behaviors.”

Conclusion

We changed “… complexity of real-world behavior …” (ll. 729-730) to “… complexity of group behavior in semi-naturalistic conditions ...”

(2) The motivation to enter the test tube is not "trait" (or at least not solely a trait) but the basic need to reach food and water; chasing behavior would be less dependent on this stimulus.

Tube traversals may reflect different motivations, including routine movement between compartments to access food, the water lickport, the open arena, or the housing area. Nevertheless, we do not interpret tube-entry motivation itself as a ‘trait’. Importantly, our hierarchy readout does not quantify which animals enter the tubes, nor is it confounded by tube-entry frequency itself (cf. ll. 527-532). Rather, it captures the consistent outcomes of incidental dyadic competitions once two animals meet in the tube (push vs. retreat), aggregated across many interactions. Thus, the stable signal we report lies in repeatable competition outcomes, not in traversal propensity. Consistent with this interpretation, the overall number of tube competition events was not associated with social rank, arguing against systematic competition avoidance by low-ranking animals. In contrast, chasing is a voluntarily initiated, asymmetric interaction between an initiator and a recipient and therefore adds a social-action component beyond incidental access-linked encounters, providing a complementary readout of social behavior.

We also noted that the term ‘trait’ may not be the optimal description in this context and replaced it throughout the ms. with more precise wording, including “internalized social rank” and “propensity to chase”.

Results

We added the following paragraph (ll. 524-532):

“Tube crossings in the NoSeMaze are motivated by the intent to eat, drink, sleep, or socialize. Accordingly, competitions within the tube arise by chance when two animals enter from opposite sides at the same time. Social rank therefore captures the consistent outcomes of repeated incidental competitions (push versus retreat). This measure is not confounded by differences in tube engagement, as social rank was neither associated with participation in tube competitions (ρ = 0.11, p = 0.139; Fig. 6C, Supplementary Fig. S12C) nor with the overall number of tube detections when controlling for chasing (Spearman’s partial correlation between detection count and z-scored David’s score, corrected for the fraction of active chases: ρ = 0.022, p = 0.758).”

Discussion

We changed the following paragraphs:

Line 608-611

“Importantly, participation frequency and differences in tube entry time did not confound the resulting social ranks, underscoring the robustness of the automated incidental rank assessment.”

Lines 653-662

“These dynamic aspects of chasing, including its asymmetric initiator–recipient structure and proactive engagement, differ from the nature of tube competitions, which are incidental encounters. Together, tube-derived social rank and chasing describe complementary dimensions of social position, and, alongside other features such as clique formation [36], contribute to describe facets of a broader multidimensional social behavior. Within this complex environment, chasing emerges as a flexible behavioral propensity that is dissociable from formal social rank. Specifically, in the NoSeMaze, chasing contributes dynamically to the maintenance, negotiation, or clarification of social hierarchy structure.”

(3) Dominance is only one aspect of sociality, social structure is reduced to rank. The information that might lie in the chasing behavior is not optimally used to explain social behavior beyond the rank measure.

In this manuscript, we focused on the relationship between social rank derived from incidental tube competitions and chasing. We agree that social rank derived from tube competitions captures only one aspect of social structure. We have now clarified this point more explicitly in the revised manuscript. Here, our specific aim was to determine how chasing relates to competition-derived social rank and whether it provides information beyond rank in these mouse groups.

We also refer to another manuscript dedicated to additional aspects of social structure captured in the video data, including approach and interaction behavior as well as social clique formation. There, these measures are again examined in relation to chasing and social rank. We also plan future studies leveraging chasing in the NoSeMaze as a readout for neurophysiological investigations.

In the present study, social rank is based on dominance and subordination in incidental competitions in the integrated tube test. We treat chasing as a distinct, volitional social dimension rather than redundant “rank information”. Importantly, our chasing analyses add beyond social rank information in three ways: (1) structural asymmetry (initiator vs recipient roles) not captured by symmetric social rank measures; (2) elite-centric reciprocal dynamics rather than broad top-down enforcement; and (3) context dependence, with social rank–chasing coupling strengthening when group transitivity is lower. We revised the Discussion/Conclusion accordingly and additionally note that other aspects of social organization (e.g., affiliative bonding and higher-order network structure such as clique/rich-club organization; Nelias et al., 2025) require complementary measures (e.g., video-derived interaction networks) and are not the scope of the present study.

To avoid ambiguity, we also added the following operational definitions in the Introduction and Results:

Introduction

Lines 90-94

“In this study, we use social hierarchy to denote the group-level structure inferred from incidental competitions in the integrated tube tests, and social rank for an individual’s level within that hierarchy. Social position serves as an umbrella term for social rank and chasing behaviors.”

Results

Line 306-308

“Here, social hierarchy refers to the group-level structure reconstructed from incidental competitions in the integrated tube tests, social rank to an individual’s position within that structure, and social position to social rank together with chasing.”

We additionally clarified the distinction between chasing and social rank in the following sections.

Discussion (ll. 632-664)

“In more constrained or despotic conditions, chasing can serve as a unidirectional, dominance-related behavior directed at subordinates [9,43,44]. The larger groups observed in the NoSeMaze reveal a more nuanced role for chasing behavior. Chasing levels are individually stable, but the expression and meaning of chasing are context-sensitive. Chasing was neither broadly distributed nor consistently directed down the social hierarchy. Instead, it was initiated by a small subset of individuals – primarily those occupying high competition-based social ranks – and frequently occurred reciprocally within this group, suggesting intra-elite social dynamics rather than broad dominance enforcement. Rather than solely serving to impose social hierarchy, chasing appeared to function as a means through which individuals with high social rank monitor, negotiate, and maintain their relative standing within the top tier. Notably, the identity of frequent chasers remained stable over time and persisted across changing group compositions, indicating that the propensity to initiate chases reflects a consistent individual-level tendency rather than a purely situational response.

However, its coupling to social rank depended on the group’s hierarchy structure. In groups with less clearly defined social hierarchies (i.e., lower transitivity), active chases aligned more strongly with social rank, suggesting that mice in less structured groups rely more on proactive signaling to clarify social rank. Indeed, the top-ranked mice in these groups exhibited relatively high levels of active chases. This context-sensitivity highlights chasing’s dual role: it serves as a tool for negotiating social rank among mice at the upper end of the hierarchy, and additionally functions to establish or reinforce hierarchical clarity when social structures are ambiguous. These dynamic aspects of chasing, including its asymmetric initiator–recipient structure and proactive engagement, differ from the nature of tube competitions, which are incidental encounters. Together, tube-derived social rank and chasing describe complementary dimensions of social position, and, alongside other features such as clique formation [36], contribute to describe facets of a broader multidimensional social behavior. Within this complex environment, chasing emerges as a flexible behavioral propensity that is dissociable from formal social rank. Specifically, in the NoSeMaze, chasing contributes dynamically to the maintenance, negotiation, or clarification of social hierarchy structure. These findings reveal a novel aspect of social dynamics: chasing is not merely a dominance display but a flexible context-dependent mechanism shaped by both individual disposition and group-level social structure.”

Conclusion (ll. 718-723)

“Crucially, social position is not fully described by a single behavioral dimension. We focused here on two separable dimensions: competition-based social rank and proactive chasing. Future work should integrate additional dimensions of social organization, such as affiliative bonding and higher-order network measures [36], to capture additional aspects of social organization. Chasing behavior played a dual role, reflecting a stable individual propensity while also adapting to group-level structure.”

(4) Focus on rank bears the risk of overgeneralization for readers not familiar with the context.

As already discussed above (see also point 2 and 3), we have sharpened the framing to reduce the risk of overgeneralization. Throughout the manuscript, we now refer to tube-derived social rank explicitly as a dominance-subordination-related axis of social organization rather than a comprehensive measure of “sociality”, and we also avoid the term “traits”, but prefer the use of “internalized social rank” or “propensity to chase” when discussing stability across rounds and contexts. Also, we removed “personality-like” as the scope of the study is to set specific behaviors in relation and not to enter the field of mouse personality classification.

We clarified the definition of social rank in this manuscript in the Introduction (ll. 90-94) and the beginning of the Results (ll. 306-308, see also point 3).

Abstract

Lines 41-46

“… Across more than 4,000 mouse-days, hierarchies derived from incidental competitions in the integrated tube tests were non-despotic, transitive, and stable even when group compositions changed. This stability supports an internalized component of competition-based social rank. Chasing was also stable across contexts. Notably, chasing was concentrated among high-ranking individuals, consistent with ongoing negotiation of social rank among individuals at the upper end of the hierarchy.”

Lines 50-54

“In summary, high-dimensional tracking with the NoSeMaze reveals that social position in mice is multifaceted and shaped by stable dimensions of individual behavior that persist across changing social contexts. The approach thus enables longitudinal modeling of individuality and social position as key resilience factors.”

Discussion (ll. 622-626)

“This temporal and contextual stability supports interpreting social rank as a stable, internalized characteristic of individuals. In this sense, ‘internalized’ refers to stability across repeated rounds and reshuffled groups in this paradigm and to relatively stable tube-competition outcomes.”

Changes in the Conclusion starting l. 709 as highlighted in answer to the above point 3.

(5) Conclusion only valid for the reductionistic setting, in which environment, social and non-social changes only within narrow limits, and in which the mouse population does not face challenges

Our conclusions are bounded by the conditions tested, namely an enriched but stable environment, controlled group composition and remixing, and the absence of explicit ecological challenges such as resource scarcity or predators. As described above in the answer to point 1, we now state explicitly that our conclusions apply to the social-context variation tested here (controlled group reshuffling) under otherwise stable conditions, and we frame resource-competition and environmental-stressor manipulations as future variation to test of how this manipulation affects the here described behaviors.

We accordingly changed the Discussion as already highlighted in point 1 above and added ll. 693-704.

(6) Animals are not naive at the beginning of the experiment, but are already several weeks old.

Animals entered the study as adults and were continuously group-housed (3-5 mice/cage) before entering the NoSeMaze. To reduce effects of initial apparatus novelty, mice underwent two NoSeMaze habituation sessions prior to data collection (each several hours). We now clarify these points explicitly in the Methods and scope the inference accordingly. One of our future steps is to extend this framework to earlier developmental stages (e.g., adolescence/weaning) to capture full lifespan trajectories. This however first required establishing the approach in adult mice under controlled conditions as done in the present study.

Methods (ll. 739-747)

“A total of 79 adult male homozygous OXTRfl/fl mice (B6.129(SJL)-Oxtrtm1.1Wsy/J, RRID:IMSR_JAX:008471, Jackson Laboratory) backcrossed > F10 to C57BL/6J background (Charles River, Sulzfeld) were used for the experiments. Animals entered the NoSeMaze as adults (see Supplementary Table S2 for ages at NoSeMaze entry across rounds) and had been continuously group-housed after weaning (3–5 mice/cage), i.e., they were not developmentally or socially naïve at study onset. Of the 79 mice, 26 mice were injected six weeks before the start of the experiment with an AAV expressing Cre recombinase (rAAV1/2-CBA-Cre) into the AON pars centralis to induce bilateral OXTR deletion (OXTRΔAON). The remaining 53 animals received an AAV expressing only dTomato (rAAV1/2-CBA-dTomato). …”

Discussion (ll. 629-631)

“… A future direction is to extend this framework to earlier developmental stages to understand which early experiences shape later trajectories of social position.”

In summary, this is a wonderful study, but not one that is easy to interpret. The bold conclusion is valid only within the constraints of the study, but nevertheless points in an important direction. The paradigm is clever and could be used for many interesting follow-ups.

To define social position as a personality trait will elicit strong opposition and much debate; the nuances of the paper might be lost on many readers and call for the (re)-consideration of many concepts that are touched. I find this attitude a strength of the paper, but the approach bears the risk of misunderstanding.

We thank the Reviewer for the helpful comments. As detailed above, we tightened terminology and framing throughout the revised manuscript by defining stability explicitly in terms of repeatability across rounds and reshuffled groups within this paradigm, and described tube-derived social rank as one dimension of social behavior. We hope these edits preserve the conceptual message while reducing the risk of misunderstanding.

**Reviewer #2 (Public review):**

Summary:

This manuscript presents the "NoSeMaze", a novel automated platform for studying social behavior and cognitive performance in group-housed male mice. The authors report that mice form robust, transitive dominance hierarchies in this environment and that individual social rank remains largely stable across multiple group compositions. They further demonstrate that social dominance and aggressive behaviors, like chasing, are partially dissociable and that dominance traits are independent of non-social cognitive performance. The study includes a genetic manipulation of oxytocin receptor expression in the anterior olfactory nucleus, which showed only transient effects on social rank.

Strengths:

(1) Innovative Methodology:

The NoSeMaze platform is a technically elegant and conceptually well-integrated system that enables fully automated, long-term monitoring of both social and cognitive behaviors in large groups of group-housed mice. It combines tube-test-like dominance contests, voluntary chase-escape interactions, and an embedded operant olfactory discrimination task within a single, ethologically relevant environment. This modular design allows for high-throughput, minimally invasive behavioral assessment without the need for repeated handling or artificial isolation.

(2) Experimental Scale and Rigor:

The study includes 79 male mice and over 4,000 mouse-days of observation across multiple group reshufflings. The use of RFID-based identification, automated data logging, and longitudinal design enables robust quantification of individual trait stability and group-level social structure.

(3) Multidimensional Behavioral Profiling:

The integration of social (tube dominance, proactive chasing), physical (body weight), and cognitive (olfactory learning task) measures offers a rich, multi-dimensional profile of each individual mouse. The authors' finding that social dominance traits and non-social cognitive performance are largely uncorrelated reinforces emerging models of orthogonal behavioral trait axes or "animal personalities".

(4) Clarity and Data Analysis:

The analytical framework is well-suited to the study's complexity, with appropriate use of dominance metrics, mixed-effects models, and permutation tests. The analyses are clearly explained, statistically rigorous, and supported by transparent supplementary materials.

We thank the Reviewer for their evaluation of our manuscript. We appreciate their recognition of the NoSeMaze as an innovative and conceptually integrated platform, the scale and longitudinal rigor of the dataset, and the clarity and appropriateness of the analytical framework.

Weaknesses:

(1) Conceptual Novelty and Prior Work:

While the study is carefully executed and methodologically innovative, several of its core findings reaffirm concepts already established in the literature. The emergence of stable, transitive social hierarchies, the persistence of individual differences in social behavior, and the presence of non-despotic social structures have all been previously reported in mice, including under semi-naturalistic conditions (e.g., Fan et al., 2019; Forkosh et al., 2019). Although this work extends those findings with greater behavioral resolution and scale, the manuscript would benefit from a clearer articulation of what is genuinely novel at the conceptual level, beyond the technological advance.

We agree with the Reviewer that transitive dominance hierarchies and stable inter-individual differences have been demonstrated previously in mice, including in semi-naturalistic settings. Our intent was therefore to address two more specific conceptual questions that prior work typically has not tested in an integrated way:

(1) Whether an individual’s social position generalizes across distinct social contexts created by systematic changes in group composition rather than reflecting stability that is only observable within a fixed group;

(2) How the two frequently employed social dominance metrics tube competition-based social rank and chasing relate to each other in unperturbed larger groups.

Specifically, the conceptual advance is enabled by (A) continuous, handling-free estimation of competition-based social rank from incidental tube contests over weeks, (B) a repeated group-reshuffling (“accelerated longitudinal”) design that explicitly tests whether an individual’s social rank generalizes across distinct social contexts, and (C) parallel, continuous measurement of chasing and non-social reinforcement-learning behaviors in the same individuals and environment.

This integration also lets us dissociate dominance-related social dimensions (competition-based social rank vs chasing) and test their relation to individual styles in non-social reinforcement-learning. Importantly, these questions are addressed in larger intact societies (9–10 mice), where hierarchy structure is shaped by more complex network-level dynamics than in smaller groups.

We revised the Introduction and Discussion to foreground these conceptual points and to position them more explicitly in the context of prior works.

Introduction

We adapted the following section (ll. 76-98) and integrated the suggested references:

“… In mice, small groups tend to form highly despotic hierarchies [24-26], whereas larger groups exhibit more complex structures [27]. These patterns suggest a strong influence of emergent group-level dynamics on social structure [12,27]. Yet, animals do not enter social groups as blank slates [28-30]; stable latent factors in the individual may also contribute to hierarchy formation [13]. Thus, it remains unclear to what extent an individual’s social position is internalized and persists across different social contexts [31], or instead is primarily an emergent property of group-level dynamics. Disentangling these possibilities requires experimental conditions that allow unperturbed, continuous tracking of all individuals in sufficiently large groups, together with systematic changes of group composition to modulate social context.

While stable hierarchies and behavioral identity domains have been described previously in semi-naturalistic settings [9-13], many studies quantify these features either within fixed group compositions or in separate assays. Here, we therefore use systematic group reshuffling in 10-member societies to directly test whether individual differences in social rank and chasing persist across distinct social groups. In this study, we use social hierarchy to denote the group-level structure inferred from incidental competitions in the integrated tube tests, and social rank for an individual’s level within that hierarchy. Social position serves as an umbrella term for social rank and chasing behaviors. By continuously measuring social rank, chasing, and reinforcement-learning behavior in the same individuals, we further test how chasing and social rank are related to each other and how they relate to individual styles in non-social reinforcement learning.

To enable these tests, we developed the Non-invasive Sensor-rich Maze (NoSeMaze).”

Discussion

We added a brief framing statement at the beginning of the Discussion (ll. 577-583) to clarify the study’s conceptual novelty:

“Ecologically enriched, yet experimentally controlled assessments allow us to study behavioral individuality and social structure in group-living animals over extended timescales. Here, we show that individual mice carry stable, individual-specific, and multi-faceted profiles of social position and cognitive styles across changing group contexts. Our approach goes beyond prior work by testing cross-context stability under repeated, systematic group reshuffling in larger mouse societies, while measuring competition outcomes, chasing, and reinforcement-learning behavior in parallel. …”

(2) Role of OXTR Deletion:

The inclusion of the OXTR manipulation feels somewhat disconnected from the manuscript's central aims. The effects were minimal and transient, and the authors defer full interpretation to a separate study.

We appreciate the Reviewer’s point and agree that the OXTR^ΔAON^ manipulation can appear secondary to the manuscript’s central aims. We included OXTR^ΔAON^ because oxytocin-dependent social recognition memory was hypothesized originally to impact potentially also learning an individual’s position in social hierarchy networks. Even though the effects were small and transient, we nevertheless believe it is relevant to report them, also in relation to the more profound effects of the genetic manipulation reported in a related manuscript (Nelias et al.,Nature Communications. 2026;17:8493, 10.1038/s41467-026-76841-5). Therefore, we explicitly account for genotype as a covariate in the analyses such that the main conclusions do not depend on this manipulation.

To improve coherence, we have added one sentence in the Introduction motivating why OXTR^ΔAON^ was included, and finally more clearly signposted that deeper mechanistic interpretation is beyond the scope of the present manuscript.

Introduction (ll. 115-120)

We added one short sentence introducing the rationale behind the perturbation:

“… and (3) determine whether social rank, chasing, and non-social reward-seeking behaviors represent stable individual characteristics or dynamic features across time and changing group composition. As a secondary analysis, motivated by oxytocin’s established role in social recognition memory [32,33], we also tested whether OXTR deletion in the anterior olfactory nucleus produces detectable shifts in rank dynamics.”

Results

Lines 161-164

“… This manipulation was included as a secondary biological perturbation. The primary analyses and conclusions focus on the platform and cross-context stability, and genotype is treated as a covariate unless stated otherwise. …”

Lines 451-454

“… As a secondary analysis, we tested whether OXTR^ΔAON^, which impairs de novo social recognition memory required for social clique formation in this cohort [36], also affects the measures reported here. Consistent with largely internalized features, OXTR^ΔAON^ produced only transient effects. …”

Discussion (ll. 594-605)

“This study focused primarily on the relation of social rank and chasing. We however also considered their relation to additional variables including the loss of oxytocin receptors in the olfactory cortex in the adult (OXTR^ΔAON^), involved in de novo social recognition learning. The propensity to chase was largely unaffected by OXTR^ΔAON^. Mice carrying OXTR^ΔAON^ displayed a transient reduction in social rank during the first week that normalized thereafter. This transient effect contrasts to the persistent impairment by OXTR^ΔAON^ in forming higher-order social bonds that enable membership in stable cliques, as identified by video tracking of self-paced interactions in the same cohort [36]. Together, these findings suggest that OXT-dependent olfactory learning is critical for the formation of social context-dependent higher-order bonds, but plays a limited role in shaping hierarchy-related behaviors.”

(3) Scope Limitations (Sex and Age):

The study is limited to male mice, and although this is acknowledged, the title and overall framing imply broader generalizability. This sex-specific focus represents a common but problematic bias. Additionally, results from the older mouse cohort are under-discussed; if age had no effect, this should be explicitly stated.

We thank the Reviewer for this relevant point. The study is limited to male mice. We therefore revised the title and the abstract and strengthened the limitations to make the sex-specific scope explicit.

We additionally note ongoing work extending the same framework to female groups, where we find similar hierarchy structure and cross-context stability in the NoSeMaze in preliminary unpublished data.

Regarding age, our design included two separate cohorts of different adult age ranges (young and older adult animals), but age was not the primary experimental factor. As detailed in our response to Reviewer #3 (point 1), we now quantify age structure explicitly and test age effects using a decomposition that separates between-group age differences from within-group age variation (mean age per group and each animal’s deviation from that mean). We also recomputed stability estimates with age-adjusted ICC models, and the resulting ICCs are highly similar to the original estimates (cf. new Supplementary Table S4), indicating that the reported metrics’ stability is not driven by age differences across groups.

Title

We change the title from “Individual differences drive social hierarchies in mouse societies” to “Individual differences drive social hierarchies in male mouse societies”

Abstract

We also added male in the abstract (ll. 37-38):

“The interaction of these behaviors in the shaping of social position in larger male mouse groups remains largely unknown.”

Discussion (ll. 613-617)

“… While this study focused on male mice, in which social hierarchies are best established [9], future work is needed to explore sex-specific expressions of social structure and their neurobiological underpinnings in female groups. We therefore restrict our interpretation to male mice. Critically, male social ranks were robustly maintained within the same group over time. …”

For a more detailed integration of age in the Methods, Results, and Discussion, we kindly refer to the reply to Reviewer #3, point 1.

(4) Ambiguity of Dominance as a Construct:

While the study robustly quantifies social rank and hierarchy structure, the broader functional meaning of "dominance" remains unclear. As in prior work (e.g., Varholick et al., 2019), dominance rank here shows only weak associations with physical attributes (e.g., body weight), cognitive strategy, or neuromodulatory manipulation (OXTR deletion). This recurring pattern, where rank metrics are reliably established yet poorly predictive of other behavioral or biological traits, raises important questions about what such measures actually capture. In particular, it challenges the assumption that outcomes in paradigms like the tube test or chase frequency necessarily reflect dominance per se, rather than other constructs.

We thank the Reviewer for this clarifying point. We agree that stable social rank metrics do not necessarily imply a complete or unitary measure of “dominance.” In the revised manuscript, we therefore clarified that, in this study, social rank is operationalized as consistent competitive outcomes in incidental tube-test encounters in the NoSeMaze (see also Reviewer #1, points 2-4).

Our data indicate that this competition-based social rank is related to, but not identical with, other social behaviors such as chasing. Likewise, body weight significantly contributes to social rank, but explains only part of the variance. We therefore do not interpret weak or partial associations with other variables as invalidating the social rank measure. Rather, we interpret them as indicating that social position is multidimensional and only partially captured by any single assay.

In independent subsequent studies that are currently in preparation or revision, we observed that heterogeneity in the neurobiology and response to challenges was best predicted by the competition-based social rank, also compared to the other behaviors assessed here. While these observations are beyond the scope of the present manuscript, they support our view that incidental tube competition and the resulting dominance-subordination structure may provide a biologically informative measure of one important dimension of social position. At the same time, the observation here and in many previous studies that factors such as body weight explain only a limited portion of the variance remains important for our understanding of these constructs.

We have revised the manuscript accordingly to make this distinction more explicit. In particular, we now define more precisely the terms social hierarchy, social rank, and social position in the context of this manuscript (see also reply to Reviewer #1, point 3; ll. 90-94 in the Introduction and ll. 306-308 in the Results), and we use these terms more consistently throughout. We also revised the text to avoid overstating the meaning of “dominance” where the data support a more specific interpretation.

Finally, we now emphasize more clearly both the strength and the limitation of the present approach. The NoSeMaze allows these relationships to be assessed continuously in a minimally perturbed group-housing ecology, laying ground for future incorporation of further variables and dimensions to capture how they shape social organization.

Specifically, we added text in the Discussion and Conclusion to clarify that competition-based social rank and proactive chasing represent separable dimensions related to dominance and subordination, but do not exhaust sociality or individuality, and that future work should integrate additional measures such as affiliative behavior and higher-order network structure.

Discussion

Lines 624-6631:

“… In this sense, ‘internalized’ refers to stability across repeated rounds and reshuffled groups in this paradigm and to relatively stable tube-competition outcomes. The tube-derived social rank describes here a dimension of individual social behavior. The capacity to quantify stable individual differences across changing social contexts highlights the value of the NoSeMaze for lifespan-oriented studies of behavioral individuality. A future direction is to extend this framework to earlier developmental stages to understand which early experiences shape later trajectories of social position.”

Conclusion

Lines 718-722:

“Crucially, social position is not fully described by a single behavioral dimension. We focused here on two separable dimensions: competition-based social rank and proactive chasing. Future work should integrate additional dimensions of social organization, such as affiliative bonding and higher-order network measures [36], to capture additional aspects of social organization.”

**Reviewer #3 (Public review):**

Reinwald et al. present the NoSeMaze, a semi-natural behavioral system designed to track social behaviors alongside reinforcement-learning in large groups of mice. Accumulating more than 4,000 days of behavioral monitoring, the authors demonstrate that social rank (determined by tube competitions) is a stable trait across shuffled cohorts and correlated with active chasing behaviors. The system also provides a solid platform for long-term measurements of reinforcement learning, including flexibility, response adaptation, and impulsiveness. Yet, the authors show that social ranking and chasing are mostly independent of these cognitive traits, and both seem mostly independent of oxytocin signaling in the AON.

Strengths:

(1) The neuroethological approach for automated tracking of several mice under semi-natural conditions is still rare in social behavioral research and should be encouraged.

(2) The assessment of dominance by two independent measures, i.e., spontaneous tube competitions and proactive chasing, is innovative and valuable.

(3) The integration of a long-term reinforcement-learning module into the semi-natural system provides novel opportunities to combine cognitive traits into social personality assessments.

(4) The open-source system provides a valuable resource for the scientific community.

Limitations:

(1) Apparent ambiguity and inconsistency in age structure and cohort participation across rounds, raising concerns about uncontrolled confounds.

(2) Chasing behavior appears more stable than tube-test competitions (Figure 4D vs. Figure 3D), which challenges the authors' decision to treat tube competitions as the primary basis for hierarchy determination.

We thank the Reviewer for the evaluation of our work. We have addressed the limitations raised below with additional analyses, clarifications, and corresponding manuscript revisions.

Major concerns:

(1) Unclear and inconsistent handling of age groups and repeated sampling. The manuscript repeatedly refers to "younger" and "older" adults, but it is unclear whether age was ever controlled for or included in models. Some mice completed only one round, others 2-5 rounds, without explanation of the criteria or balancing.

We thank the Reviewer for this clarifying comment. We now explicitly quantify participation in the different rounds in new Supplementary Table S3. Importantly, our primary stability analyses are implemented using variance-component mixed models (REML) that naturally handle unbalanced repeated-measures data. This is explained in more detail in the Methods section (ll. 1003-1111), where we added a statement that the LMEs are well suited for unbalanced repetitions.

To further address unbalanced participation, we additionally performed a conservative sensitivity analysis restricted to a balanced subset, including only sessions 1 and 2 and only mice with observations in both sessions. For the key social measures, ICC estimates were highly similar in the full dataset and in the balanced subset (e.g., z-scored competition David’s score, ICC across cohorts: 0.55 without age adjustment vs. 0.56 in the balanced first-two-session subset; active chasing: 0.74 vs. 0.72; being chased: 0.61 vs. 0.60; Supplementary Table S4), indicating that unbalanced participation did not inflate the stability estimates.

We also addressed age structure explicitly. Although age was not the primary experimental factor, the inclusion of two age cohorts allowed us to assess whether the observed behaviors and their interrelations were robust across most of the adult lifespan (cf. new Figure 1). We therefore decomposed age into a between-group component (mean age per group) and a within-group component (each animal’s deviation from its group mean) and included these terms in the relevant LME models. Tube-based dominance rank (David’s score, z-scored) showed no age effect (p_age, cond._ = 0.63), whereas chasing metrics showed modest age associations (cf. new Supplementary Table S5). This however only indicates that chasing was associated with age to some degree. More importantly, recomputing all stability estimates using age-adjusted ICC models yielded nearly identical ICCs for the core social measures (new Supplementary Table S4), indicating that the reported stability was not affected by age differences across groups.

In addition, we revised the study design schematic (new Fig. 1; cf. Reviewer #1, Recommendations for the authors) to depict the separate age cohorts and the reshuffling procedure more clearly.

We adapted the following sections accordingly.

Methods

Lines 980-985

“… Most mice (n = 68) participated in at least two NoSeMaze rounds with reshuffled group members. Supplementary Table S2 summarizes the number of rounds per mouse and missing data due to technical problems. We examined the stability of social and reward-seeking metrics by correlating values from the first and second round (Spearman’s correlation, cf. Fig. 5). These round-1-to-round-2 correlations use one paired observation per mouse and are therefore not inflated by mice contributing >2 rounds.”

Lines 991-993

“The ICC treated mouse identity as a random intercept and NoSeMaze group (i.e., round-specific social group) as a random effect, with repetition included as fixed effect (i.e., stability across groups while holding repetition means constant).”

Lines 1003-1011

“Variance components for ICC estimation were obtained from LMEs fit by restricted maximum likelihood (REML), which yields less biased variance-component estimates and is well suited for unbalanced repeated-measures designs (i.e., different numbers of rounds per mouse). To assess potential confounding by age structure, age was decomposed into a between-group component (group-mean age) and a within-group component (each animal’s deviation from its group mean) and included as covariates. ICCs were recomputed in age-adjusted models (see Supplementary Table S4). Finally, we performed a conservative sensitivity analysis restricted to the first two sessions per mouse and to mice with observations in both sessions (“balanced first2”), to additionally account for unbalanced participation structure.”

Results

Lines 167-173

“… The study population comprised two age cohorts: younger (16-30 weeks) and older adults (55-97 weeks) (Fig. 1B). The two age cohorts were run as separate experimental series, and group reshuffling was performed within each cohort (Fig. 1C, see Supplementary Table S2). Mice lived in groups of 9-10 for multiple rounds in the NoSeMaze, with different group members in each round (Fig. 1D, see Supplementary Table S1). This allowed us to test which individual behaviors were stable across different group compositions. …”

Lines 441-450

“Because the number of rounds in the NoSeMaze was unbalanced between animals (Supplementary Table S3) and age varied across groups (Supplementary Table S1-2), we also performed robustness checks. Stability estimates changed only minimally when recomputed in age-adjusted ICC models (between-group mean age and within-group age deviation; see Methods) and when restricting analyses to a balanced first-two-session subset (sessions 1–2 only; mice with both sessions) (Supplementary Table S4). Mixed models indicated that some chasing and reinforcement-learning measures showed modest age- and/or session-related shifts in absolute levels (Supplementary Table S5), but importantly, these did not affect the observed stability patterns.”

(2) Stability of chasing appears stronger than the stability of tube competitions. Figure 4D shows highly consistent chasing behavior across weeks, while Figure 3D shows weaker and more variable correlations for tube-based David scores. This is also evident from Figure 5A-B,D. Thus, it appears that chasing, which serves to quantify dominance in similar semi-natural setups, may be a more reliable and behaviorally meaningful measure of dominance than the incidental tube competitions.

Indeed, active chasing showed higher cross-round correlations than tube-derived social rank (e.g., R1–R2 Spearman ρ = 0.75 vs 0.57; ICC_across cohort_ 0.74 vs 0.55, see Fig. 5 and new Supplementary Table S4). Importantly, this does not contradict the central finding that tube-derived social rank is stable across time and across remixed groups. Rather, it may highlight that chasing and social rank capture different aspects of dominance-subordination-related behavior with different statistical properties. Chasing reflects an individual’s propensity to actively initiate interactions (a strongly expressed, asymmetric behavior), which can be highly consistent across contexts. By contrast, social rank is a relational measure inferred from symmetric dyadic win–loss outcomes based on incidental competitions in the integrated tube tests and can vary with the specific set of competitors and interaction opportunities in each reshuffled NoSeMaze group, while still remaining substantially stable overall.

Accordingly, we do not interpret the higher repeatability of chasing as evidence that it is the “better” hierarchy measure. Tube competitions yield symmetric dyadic outcomes that directly support formal hierarchy reconstruction (David’s score/Elo, transitivity, steepness) and show convergent validity with traditional tube testing. Chasing, in contrast, is asymmetric and volitional and, in our data, is concentrated in the upper social ranks and modulated by group-level social hierarchy structure, consistent with rank negotiation/signaling rather than a mechanism that assigns a full ordering to all individuals. These different properties lead to very different “win-lose” relationships when comparing tube competition events to chasing events that we specifically illustrated in Supplementary Fig. S13. We therefore revised the Results and Discussion to frame tube-derived social rank and chasing as complementary social dimensions.

Results (ll. 402-413)

“… Specifically, stability was high for the tube competition-based David’s score (Fig. 5A, ρ = 0.57, p < 0.001), as well as for the fraction of active chases (Fig. 5B, ρ = 0.75, p < 0.001) and of times being chased (Fig. 5C, ρ = 0.54, p < 0.001). Notably, the fraction of active chases showed slightly higher across-round stability than tube-derived David’s score and the fraction of being chased. This is in line with active chases capturing an individual propensity to initiate this behavior, whereas competition-based social rank is a relational measure that is also influenced by the set of competitors and interaction opportunities in each reshuffled group. Nonetheless also social rank and being chased were overall stable. Across the full series, ICCs for these social measures were also in the good–excellent range, indicating high across-round stability (Fig. 5D, ICC = 0.548 to 0.890).”

Discussion

Line 653-664 (see also Reviewer #1, point 2)

“These dynamic aspects of chasing, including its asymmetric initiator–recipient structure and proactive engagement, differ from the nature of tube competitions, which are incidental encounters. Together, tube-derived social rank and chasing describe complementary dimensions of social position, and, alongside other features such as clique formation [36], contribute to describe facets of a broader multidimensional social behavior. Within this complex environment, chasing emerges as a flexible behavioral propensity that is dissociable from formal social rank. Specifically, in the NoSeMaze, chasing contributes dynamically to the maintenance, negotiation, or clarification of social hierarchy structure.”

(3) Unbalanced participation across rounds compromises stability analyses. Stability analyses (e.g., ICCs, round-to-round correlations) assume comparable sampling across individuals. However, some mice contribute 1 round, others 2, 3, 4, and even 5 rounds. This imbalance may inflate stability estimates or confound group reshuffling effects, and the rationale for variable participation is not explained.

We thank the Reviewer for raising this point. Indeed, participation was unbalanced across rounds (see the same Reviewer #3, Major concerns 1 and new Supplementary Table S3), mainly due to missing data from occasional technical failures of the RFID detectors or the reinforcement learning water port during acquisition in some groups (for details, see Supplementary Table S2). We added this rationale behind variable participation to our Methods section (for details, see Major concern 1, ll. 981-982, “Supplementary Table S2 summarizes the number of rounds per mouse and missing data due to technical problems.”)

We now quantify round participation (new Supplementary Table S3) and directly address potential bias from unequal sampling in two ways. First, the round-1-to-round-2 (R1–R2) stability correlations use one paired observation per mouse and therefore are not inflated by mice contributing more than two rounds. Second, ICCs were estimated from REML variance-component mixed models that account for unbalanced repeated-measures by design and use all available observations. To further rule out inflation from unequal sampling or non-random missingness, we additionally report a conservative sensitivity ICC restricted to a balanced subset including only each mouse’s first two observed sessions and only mice with both sessions (“first2-balanced”). Full-sample ICCs and first2-balanced ICCs were highly similar (new Supplementary Table S4), indicating that participation imbalance did not affect the stability estimates. For details on the changes made in the manuscript, see Reviewer #3, Major concern 1.

**Recommendations for the authors:**

**Editor's notes:**

Should you choose to revise your manuscript, if you have not already done so, please include full statistical reporting including exact p-values wherever possible alongside the summary statistics (test statistic and df) and, where appropriate, 95% confidence intervals. These should be reported for all key questions and not only when the p-value is less than 0.05 in the main manuscript.

Readers would also benefit from noting that the mice were male in the abstract.

We have revised the manuscript accordingly and now report exact p-values values wherever possible for the key results in the main text. Because full statistical reporting for every analysis in the main text would substantially interrupt readability, we provide the complete statistical details in a Supplementary Excel File (Supplementary Material – Systematic Statistical Reporting), including sample sizes, degrees of freedom, the number and type of permutation tests, exact p-values, and 95% confidence intervals. We also provide Extended Data Sheets for all linear-mixed effects models that account for covariates such as age and round of participation in the NoSeMaze (cf. Reviewer #3, Major Concern (1) for the additional analyses). To guide readers to these resources, we now explicitly refer to the Supplementary Material – Systematic Statistical Reporting at several points in the manuscript:

Results

Lines 271-274

“Detailed statistical reporting for all analyses, including n, degrees of freedom, exact p-values, and 95% confidence intervals, is provided in the Supplementary Material - Systematic Statistical Reporting. Extended Data includes additional linear mixed-effects models controlling for potential confounding variables.”

Lines 430-431

“All statistical details are provided in the Supplementary Material – Systematic Statistical Reporting.”

Additional references to the supplementary statistical reporting were inserted at lines 252-254, 562-563, and 567-568.

Methods

Lines 962-965

“Full details on the statistical tests, including n, degrees of freedom, exact p-values, and 95% confidence intervals, are provided in the Supplementary Material – Systematic Statistical Reporting, as well as in the Extended Data for the LMEs accounting for different covariates.”

We also revised the abstract and the title to explicitly state that the mice were male.

Manuscript changes:

Results, figure legends, and supplementary tables expanded to include full statistical reporting; abstract revised to specify male mice.

**Reviewer #1 (Recommendations for the authors):**

I would recommend being much more explicit about the reductionistic nature of the study and how the limitations are turned here to an advantage, while at the same time acknowledging the challenges of extrapolating beyond these boundaries. Most importantly, dominance should be positioned more clearly and cautiously within a framework of social behavior (and social structure) in general. The authors include cognitive tests, etc., to generate context, but this context is dependent on the same circumstances that possibly contribute to the social structure. The information that lies in the chasing behavior as an additional measured variable might be used better to provide more context.

We thank the Reviewer for these recommendations. We have better clarified the framing to make explicit that the NoSeMaze is a controlled laboratory group-housing system. The specific conditions are now discussed in more detail in relation the observed social behaviors. We describe the tube-derived social rank as one dimension of social organization, and proactive chasing as another one. This study presents a first necessary step to understand their shared and distinguishing features. We also elaborated the Discussion to better clarify the trade-off between ecological complexity and experimental control, and to highlight that the value of the NoSeMaze for continuous, observer-independent phenotyping under standardized conditions. We now clearly state the importance to vary conditions in the system to see how the social behaviors and also their relation to non-social features changes depending on context conditions. For detailed changes, see our responses to Reviewer #1, points (1), (3), (4), and (5), and Reviewer #2, Weakness (4).

Manuscript changes:

Abstract, Introduction, Discussion, and Conclusion revised to clarify scope, construct interpretation, and the complementary roles of competition-based social rank and chasing.

The precision of the description of the experimental design should be improved. When were the cohorts mixed, or did they stay separate? Which animals were old, which were young? This remained a bit confusing.

We revised the presentation of the study design accordingly. Specifically, we clarified that the younger and older adult cohorts were run as separate experimental series and that group reshuffling occurred within, but not across, these cohorts. We also revised the study schematic in Fig. 1 and the corresponding description in the manuscript to more explicitly depict cohort structure, timing of NoSeMaze rounds, and between-round reshuffling (details are provided in the Supplementary Tables 1-3). For new age-related analyses and additional robustness checks, see our response to Reviewer #3, Major Concern (1).

Manuscript changes:

Figure 1 and its legend were revised to depict cohort structure, NoSeMaze rounds, and between-round reshuffling more explicitly. In addition, the Results subsection “Ecological longitudinal assessment in the NoSeMaze” were updated to clarify that the younger and older adult cohorts were run separately, that reshuffling occurred within but not across cohorts, and which animals belonged to each age-defined cohort. We also now cross-reference Supplementary Table S2 for round-specific age information and cohort composition.

Results

Lines 167-170

“The study population comprised two age cohorts: younger (16-30 weeks) and older adults (55-97 weeks) (Fig. 1B). The two age cohorts were run as separate experimental series, and group reshuffling was performed within each cohort (see Supplementary Table S2).”

It is also not fully clear across which groups stability measures were obtained: are these across all groups or within the subgroups with a given characteristic?

We thank the Reviewer for highlighting this point. We now state explicitly that stability measures were computed across all eligible rounds, using mixed-effects models that account for repeated observations of the same mouse and for round-specific group membership. We also added a conservative sensitivity analysis restricted to a balanced first-two-session subset. Full and balanced-subsample stability estimates were highly similar, indicating that the main conclusions are robust to the participation structure. For details, see our response to Reviewer #3, Major Concerns (1) and (3).

Manuscript changes:

Methods and Results revised to clarify the level of analysis and model structure, as well as new supplementary robustness tables (Supplementary Tables 3-5) added.

Minor point: In Figure 3, 18 groups are mentioned, but 19 are shown.

We thank the Reviewer for this clarification. The apparent discrepancy arose because the group labels in Figure 3 follow the numbering of all experimental groups, whereas only groups with available tube-competition data are shown in this panel. Accordingly, group 16 is absent because no tube data were available for that group (see Supplementary Table S2), and groups 20 and 21 are likewise not included for the same reason. We have revised the figure legend and corresponding text to make this explicit and to avoid the impression of a numbering inconsistency.

“Fig. 3: Social rank derived from incidental competitions in the integrated tube tests of the NoSeMaze.”

“C, Box plots of metrics characterizing social hierarchy for 18 groups, including transitivity, steepness, stability, and uncertainty-by-repeatability (for details, see ‘Source Data’). Group labels correspond to original experimental group IDs. Only groups with available tube-competition data are shown. Therefore, numbering is non-consecutive (e.g., groups 16, 20, and 21 are absent; see Supplementary Table S2).”

**Reviewer #2 (Recommendations for the authors):**

(1) To better distinguish this study from previous literature, we recommend incorporating a more focused discussion (or adding to the intro) of how the findings advance our understanding of social hierarchy beyond prior works.

We have sharpened the conceptual framing in both the Introduction and Discussion. In particular, we now distinguish more explicitly between the well-established observation that hierarchies form and remain stable within fixed semi-naturalistic groups, and the more specific question addressed here: whether an individual’s social position generalizes across changing social contexts created by repeated group reshuffling. We added additional references and also emphasize that the present study integrates continuous measurements of competition-based social rank, chasing, and reinforcement-learning features in the same individuals and environment. For details, see our response to Reviewer #2, Weakness (1).

Manuscript changes:

Introduction and Discussion revised to foreground conceptual novelty relative to prior semi-naturalistic work.

(2) Given the weak associations between dominance rank and other traits such as body weight, cognitive performance, and oxytocin receptor manipulation, we suggest further clarifying what is being captured by these measures.

We agree and have clarified this point throughout the manuscript. We now define tube-derived social rank explicitly as an operational measure based on repeated competitive outcomes from incidental dyadic tube tests, highlighting it as one dimension of social behavior.

We also make clearer that proactive chasing is not redundant with rank, but instead captures a distinct, partly dissociable dominance-related interaction mode. For details, see our response to Reviewer #2, Weakness (4).

We understand the question on the meaning of social rank as the correlations to body weight and cognitive performance are only punctual. We would like to mention here already that the competition-based social rank turns out to be a strong predictor of individual reactivity in a series of challenges. These works are in currently in preparation for publication and will make the relevance of these measures more clear. Related to this, we find in these studies that chasing and competition-based social rank predict different behavioral and neuronal aspects of individual reactivity.

Manuscript changes:

Abstract, Results, Discussion, and Conclusion revised to clarify construct interpretation and multidimensionality.

(3) We recommend modifying the title and abstract to more clearly reflect the male-only design of the study. In addition, please indicate whether any age-related differences were observed. If age had no measurable effect, this should be stated explicitly to justify the combination of age groups.

We revised the title and abstract to make the male-only design explicit. We also now report age-related analyses directly in the manuscript. Briefly, age was modeled by separating between-group age structure from within-group age variation. Some measures showed modest age associations in mean level, but most importantly, age-adjusted stability estimates for the core social metrics were highly similar to the original estimates, indicating that the reported stability is not driven by age differences across groups. For details, see our response to Reviewer #3, Major Concern (1).

Manuscript changes:

Title and abstract revised. Methods, Results, and supplementary robustness analyses expanded to report age effects explicitly.
